# Techniques and Instruments for Assessing and Reducing Risk of Exposure to Nanomaterials in Construction, Focusing on Fire-Resistant Insulation Panels Containing Nanoclay

**DOI:** 10.3390/nano14181470

**Published:** 2024-09-10

**Authors:** Romeo Cristian Ciobanu, Mihaela Aradoaei

**Affiliations:** Department of Electrical Measurements and Materials, Gheorghe Asachi Technical University, 700050 Iasi, Romania; mihaela.aradoaei@academic.tuiasi.ro

**Keywords:** nanomaterials, nanoclays, fire-resistant panels, inhalation exposure, dermal exposure, decision trees, safeguard scenarios

## Abstract

The paper explains how nano exposure is assessed in the construction field and focuses on the production of fire-resistant insulation panels with nanoclay. Utilizing the commercial ANSYS CFX^®^ software, a preliminary theoretical simulation was conducted on nano exposure in the workplace, which revealed that particle dispersion is primarily driven by diffusion. Panel post-processing through drilling results in the highest inhalation exposure, followed by mixing and grinding activities. Compared to a state of ‘no activity’, each activity resulted in an exposure increase by a factor of min. 1000. An overall assessment suggests that the use of nanoparticles in construction materials may not significantly heighten workers’ exposure to nanopowders when considering particle concentration alone as opposed to using traditional micro-scale materials. However, the issue persists when it comes to blending powders or performing finishing tasks on panels, with concentration levels being significantly higher for drilling, grinding, and mixing powders at 2.4 times above the standard reference value (40,000 particles/cm^3^); this is unacceptable, even for brief durations. Examination of dermal contact with gloves and masks worn by workers revealed no nanoparticle penetration. Safety measures were proposed for workers based on decision trees to enhance their safety. Ten categories of protection strategies have been devised to combat the impact of nanoparticles, which are tailored to specific technical situations, but they must be modified for various types of nanoparticles despite potential shared health implications.

## 1. Introduction

The utilization of nanomaterials and nanotechnologies, as defined by 2011/696/EU [1], is increasingly prevalent in various fields such as high-tech (electronics, medicine, energy sources, sensors), as well as in traditional sectors like automotive, cosmetics, sports equipment, light industry, and construction. The utilization of nanotechnology in construction is a modern method. It requires ample raw materials and mass production. However, it enables the development of innovative construction materials with enhanced properties. In comparison to the overall growth of nanomanufacturing on a global scale, the market presence of nanoproducts in the construction sector is still viewed as a specialized market segment [2]. The construction industry in Europe has been limited in its use of nanoproducts due to costs and hesitation about their long-term performance. Carbon nanotubes, nanofibers, and fullerenes, metal oxide nanopowders like SiO_2_, Fe_2_O_3_, and TiO_2_, metal nanopowders such as Cu and Ag nanopowders, and nanoclays are the most commonly used nanomaterials in the construction industry [3,4,5,6,7]. Nanotechnology in construction mainly targets four sectors: (1) Cement-based materials; (2) Noise and thermal insulation or temperature control; (3) Surface coatings for enhancing material functionality; (4) Fire protection [8,9,10,11,12]. Concrete–cement-related products made up 12% of the total nanoproducts, while insulation products accounted for 7% [8].

In recent years, there has been an increase in the nanoclay market, particularly in the construction sector, as these naturally derived nanomaterials are more cost-effective and versatile for mass production compared to synthetic carbon nanomaterials or metallic oxides, which are expensive and have limited uses [13,14,15,16,17,18]. Nanoclays are tiny particles made up of mineral silicates mixed with polymers in order to create nanocomposites. Similar to other nanoscale substances, nanoclays may exhibit harmful effects that are not present in materials with typical particle sizes. One instance of nanoclay utilization is seen in prefabricated panels containing nanoclay that provide fire resistance. Fireproof panels are pre-made components designed for use in building walls, ceilings, floors, and roofs. They offer consistent and improved insulation in comparison to conventional construction solutions and methods.

Around 75% of employees and employers in the construction industry are unaware that they are using nanomaterials in their work. There is usually a lack of thorough information on the composition of products and their potential health and safety concerns at the nano level. The data available from raw material manufacturers are frequently lost as such material moves down the supply chain to users. Due to this fact, regular construction firms struggle to perform a thorough risk evaluation and establish a secure working environment for their staff [19]. However, it is evident that the construction industry employs workers with lower levels of education, who are more vulnerable to risky situations and are less skilled in recognizing and managing these risks when compared to high-tech sectors. Workers can be exposed to nanoparticles during production, handling, cutting, packaging, and transportation, as well as during processing. In the construction sector, the group of workers most vulnerable to exposure are those who handle materials that contain nanoparticles. Inhalation, dermal, oral, and ocular routes are all ways in which people can come into contact with manufactured nanomaterials [20,21,22].

The biggest threat to health comes from inhaling dust produced by nanomaterials during activities like cutting, grinding, drilling, or machining. Elements that influence exposure to manufactured nanomaterials include the quantity of material utilized, level of concentration, and length of usage. Currently, there is a lack of occupational exposure limits for nanomaterials. At present, there is a lack of data on how products with nanoparticles are being used and the potential for exposure to nanoparticles in the workplace. There are currently no universally accepted standards or methodologies for measuring workplace performance. The primary challenge lies in the precise analysis of airborne particles in the workplace, given that nanomaterials possess distinctive physicochemical properties that set them apart from traditional materials. There is discussion in the literature regarding which parameters are important for assessing nanoparticle exposure. In workplace and laboratory studies, particle concentration and particle size distribution values are frequently utilized; however, a significant limitation of measuring instruments is their inability to distinguish between nanoparticles [23,24,25,26,27,28].

To reduce respiratory and skin exposure at work, various protective measures like substitution, technical and organizational measures, and individual protection must be implemented following the precautionary principle and good practice in occupational hygiene to minimize dust exposure. The risk management plan should have instructions for setting up and assessing engineering tools (such as exhaust ventilation and dust collection systems), teaching and training employees on the correct handling of nanomaterials (for example, following good work habits), and picking and using personal protective gear (like clothing, gloves, and respirators).

There is a scarcity of data for creating informed exposure scenarios for the entire life cycle of nanomaterials, which is produced for established purposes. The bulk of available quantitative exposure data pertains to the small-scale manufacturing of nanomaterials. Limited information exists regarding the exposure of end users, including consumers and users of products that contain manufactured nanomaterials [29]. Paying attention to particles of all sizes is crucial since nanoparticles have a tendency to cluster together to form larger particles. The nanoclay-type nanomaterial is the main focus of the exposure scenarios examined in this paper.

Various researchers have studied the health impacts of bentonite clay and kaolin, which are commonly used in the construction industry. Bentonite is made up of clay that is colloidal, very plastic, and primarily composed of montmorillonite. Kaolin is composed of various minerals, with kaolinite being the primary constituent. Characterizing clay-type materials has been difficult due to the significant variation in their composition. The level of crystalline silica, a common component in clays, has frequently been the determining factor in the toxicity caused by clay. In conclusion, there currently are limited data on the potential cancer-causing or DNA-damaging effects of bentonite clay and kaolin. According to research findings, prolonged exposure to kaolin can result in pneumoconiosis, although the likelihood is significantly lower compared to exposure to quartz [30]. Kaolin is viewed as more harmful to humans than bentonite.

Currently, there is a scarcity of data regarding the potential toxicity of nanoclay. When looking at toxicological data, it is crucial to keep in mind that the physicochemical properties can differ among materials as well as test systems (such as dosing, in vitro/in vivo cell cultures, mice/rats, and treatment duration), which are often diverse, making result comparison quite difficult. As numerous nanoclay variations are altered for nanocomposite formation, it is crucial to determine if the modification or the nanoclay itself is responsible for potential toxic effects. Further research is necessary to validate the potential lung toxicity of nanoclay through repeated inhalation studies [31].

Below, certain factors related to the evaluation of toxicity in the event of exposure to nanoclays are discussed:

1. Acute toxicity—A study in Sprague-Dawley rats found that nanosilicate platelets (TNS) from natural montmorillonite clay have low toxicity (LD50 > 5700 mg/kg body weight) [32].
2. Irritability and sensitization—Lack of information in the literature.
3. Repeated Dose Toxicity—We did not find any studies on repeated dose inhalation. In [33], the toxicity of sepiolite nanoclay was studied in male rats through intratracheal instillation. The results showed that the substance could cause temporary neutrophilic reactions 24 h after exposure, as well as the occasional formation of multinucleate giant cells at 1 week, 5 weeks, and 3 months after exposure. Furthermore, there was inflammation in the centroacinar areas 24 h after exposure, but the severity of the effects lessened as time passed.
4. Genotoxicity—There was no mutation observed in the Ames assay or ROS production in an acellular test system after exposure to unmodified nanoclay (Cloisite Na + R) and organically modified nanoclay (CloisiteR 30B) [34]. Nonetheless, both unfiltered and filtered CloisiteR 30B particles (with larger nanometer particles eliminated) caused DNA strand breaks in Caco-2 (human colon cancer) cells in a dose-dependent manner 24 h post-exposure. The study’s finding was that the organo-modifier, not the particles, was responsible for the observed DNA damage. In [35], it was mentioned that incorporating 2.5% or 5% fetal calf serum (FCS) in an unaltered nanoclay (Cloisite Na + R) dispersion medium can impact toxicity study outcomes. It was observed that the medium with 2.5% FCS or without FCS notably hindered cell growth in human U937 monocyte cells, whereas the medium containing 5% FCS had minimal impact on cell growth. The genotoxicity of PNS from natural montmorillonite clay was examined in vitro and in vivo, revealing no DNA damage in CHO cells and no significant micronucleus induction in ICR mice or mutations in the gene mutation assay [32].
5. Carcinogenic effects—No data found in the literature.
6. Reproductive toxicity—No data found in the literature.
7. Cytotoxicity—Nanoclay has been employed in making bio-nanocomposites with chitin-based polyurethane (PU) by utilizing chitin, Delite HPS bentonite nanoclay with montmorillonite, 4,4′-diphenylmethane diisocyanate, and polycaprolactone polyol CAPA 231. The impact of nanoclay content relating to mechanical properties and in vitro biocompatibility was studied. L-929 testing showed that the optimal PU bio-nanocomposite included 2% Delite HPS nanoclay with bentonite. Higher nanoclay concentrations led to elevated cytotoxicity in fibroblasts [36]. Researchers examined how unaltered nanoclay (Cloisite Na + R) and modified nanoclay (Cloisite 93AR) affected human HepG2 hepatoma cells in vitro and found both types to be extremely toxic, with Cloisite Na + R specifically triggering reactive oxygen species (ROS) production [37].

The study aimed to evaluate inhalation and skin exposure in the building materials industry, highlighting the potential risk for workers despite standard ventilation. The simulation demonstrated that the particle transport equation adequately explains the issue of nano exposure and can be replicated by any interested party using the same software to analyze any specific manufacturing site. The paper innovatively describes the assessment of nano exposure in the construction industry, specifically focusing on the production of fireproof insulating panels with nanoclay, which can be further particularized for other specific nanomaterials used in the building industry. It also includes safety measures for workers by developing specific decision trees, which can be further generalized for other specific technological processes in building areas.

## 2. Materials and Methodology

### 2.1. Nanoclay as Nanomaterial Ingredient

The study focused on evaluating workers’ exposure to nanopowders generated from bentonite during the production of fire-resistant panels. The research utilized natural nanoclay bentonite activated with 5–10% sodium carbonate to enhance the ion exchange mechanism.

**Figure 1:**
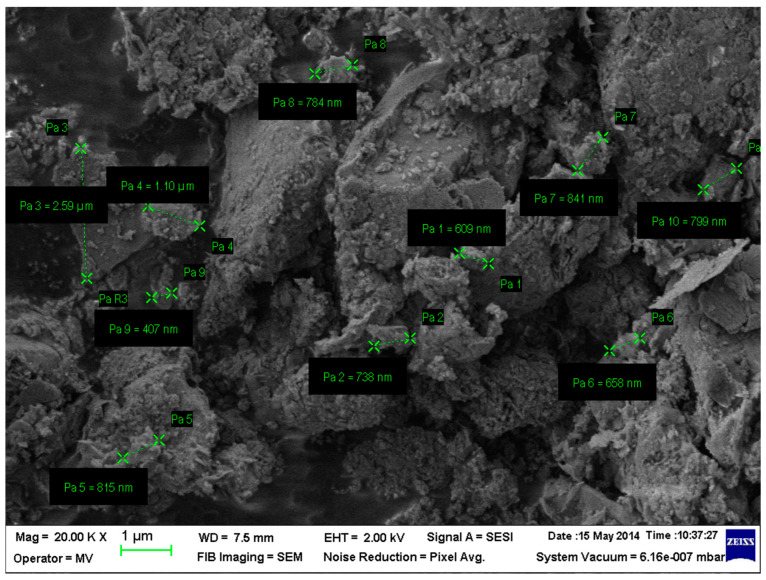
SEM image and particle size of bentonite nanoclay.

**Figure 2:**
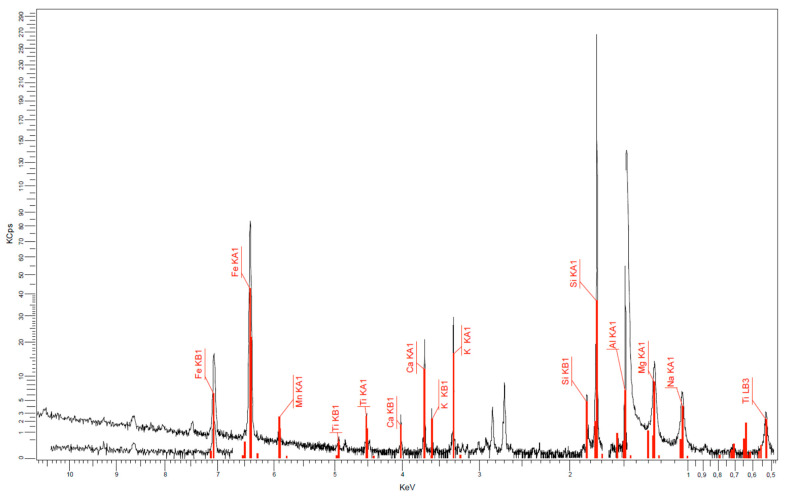
Composition of bentonite nanoclay.

The microscopic image and bentonite nanoclay composition are presented in Figure 1 and Figure 2 and Table 1 (using Lyra III XMU equipment—TESCAN GROUP a.s., Brno-Kohoutovice, Czech Republic).

The formula for bentonite used in our experiments (a mixed Ca/Mg/Na/K bentonite) is as follows (1):

(Al_1.67_Mg/Ca_0.33_)[(OH)_2_|Si_4_O_10_] · Na/K_0.33_(H_2_O)_4_ (1)

### 2.2. Theoretical Simulation of the Nano Exposure at Working Place

**Figure 3:**
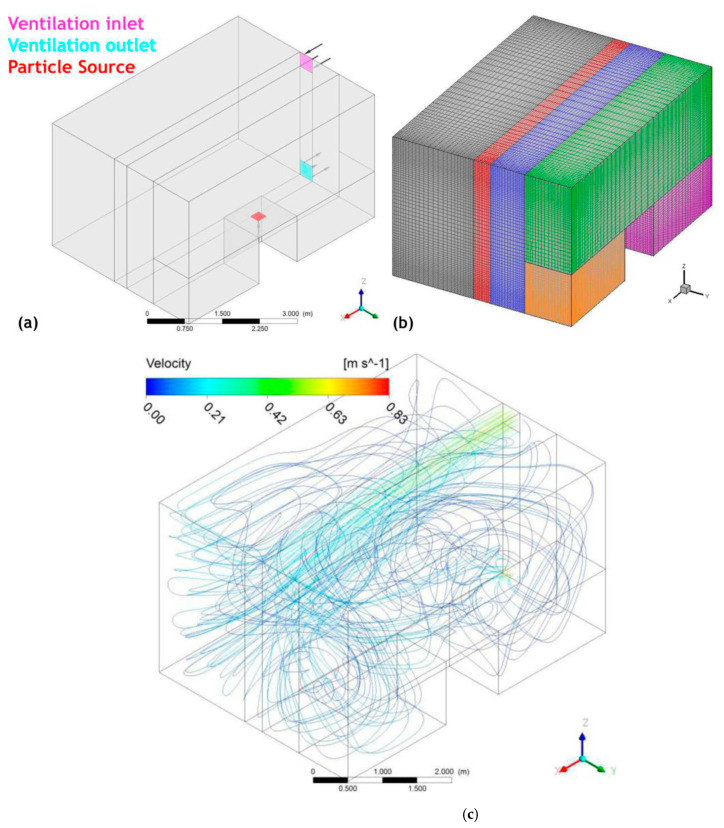
The simplified geometry of chamber (**a**), related grid (**b**), and streamlines of the air field within chamber (**c**).

The modeling process in three steps involves using the commercial ANSYS CFX^®^ software [38]: first, create the geometry and grid for the space of interest, then calculate air properties and turbulence, and finally, use the air flow field to analyze nanomaterial dispersion in space with the CFD-based program mentioned in [39] and ANSYS CFX^®^. Utilizing a comprehensive Eulerian model, the study considered particle convection, Brownian diffusion, particle inertia, and gravitational settling. The method was commonly utilized to evaluate the occupational exposure to engineered nanomaterials in a practical work setting, first by assessing how the nanomaterials spread in a simulated area with basic shapes and then confirming the idea at an actual production site for nanomaterial-based panels. The simplified production space’s geometry is shown in Figure 3. The measurements are L 5.25 m × W 3.85 m × H 3 m, and the desk measurements are L 1 m × W 1 m × H 1.1 m (smaller size, as per ideal software design). The ventilation holes for intake and exhaust are square-shaped and measure 35 cm on each side, Figure 3a). The arrangement of the calculation grid consists of 6 blocks clustered near the walls. The commercial package ANSYS Meshing of ANSYS CFX^®^ was used to generate and validate the structured computational grid. A grid independence test was performed, the result remained stable starting from approx. 332,000 nodes, and the model presented in the study is based on these figures, Figure 3b). The simulation parameters are isothermal flow at 25 °C, uniform air speed at the entrance, air flow at the entrance through the ventilation hole: Q = 0.06125 m^3^/s (~220 m^3^/h), constant average static pressure of 0 atm at outlet, and k-ε turbulence model.

**Figure 4:**
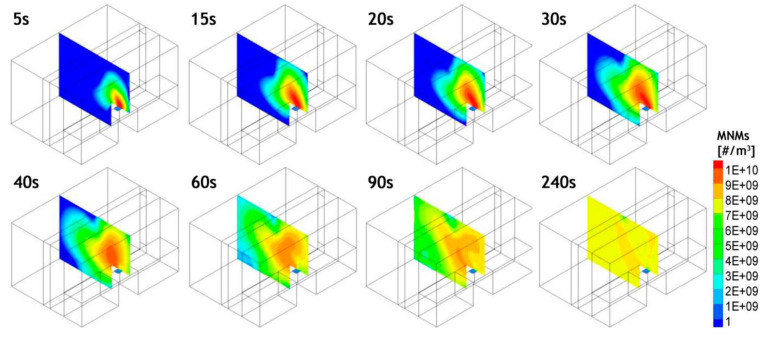
Concentrations of nanomaterials over time in the median plane of the space.

Figure 3c displays the streamlines of the air field. The flow field is intricate in the vicinity of the working table; however, air speed is decreased in all parts of the room. The particle transport Equation (2) explains how nanomaterials spread out in three-dimensional space.

(2) $$\frac{\partial n}{\partial t}+\nabla \cdot \left(n\vec{u}\right)=\nabla \left[\frac{\mu_{eff}}{\sigma_{t}}\nabla n\right]+S_{n}$$

where $n\left(\vec{r},t\right)$—the particle number concentration at the point $\vec{r}$ at time *t*, $\vec{u}$ is the air flow velocity field, and *S_n_* is the particle source; $\mu_{eff}$—effective viscosity and $\sigma_{t}$—turbulent diffusivity. The simulation hypothesis is as follows: initial background concentration of manufactured nanomaterials—zero; the nanomaterials (dimension > 10 nm) are released from a flat source (23 × 23 cm), with a speed of 0.1 m/s; the release model of nanomaterials is that of a step function, with an equivalent in free space of 10^10^ particles/m^3^; the emission of nanomaterials is stopped after 30 s; and the total simulation time is 4 min. The simulation results as concentrations of nanomaterials over time are briefly presented in Figure 4.

Expanding the simulation of nanomaterial dispersion to consider inertial and gravitational effects of particles, as performed in [39], showed that these mechanisms do not significantly impact the geometry of the space being studied, even with larger particles. Therefore, the particle transport equation provided adequately explains the issue of nano exposure. It was observed that the concentration of nanomaterials in the worker’s breathing zone stabilizes at a fairly constant value two minutes after the emission is stopped. Because diffusion is the main way particles spread out, the decrease in concentration afterward occurs gradually. The anticipated average exposure after 2 min was calculated to be 5.24 × 10^8^ particles/m^3^, a highly worrisome figure in regard to health hazards, to be contrasted with actual measured values at the production facility under similar circumstances.

### 2.3. Manufacture of Fire-Resistant Panels in Real Conditions

**Figure 5:**
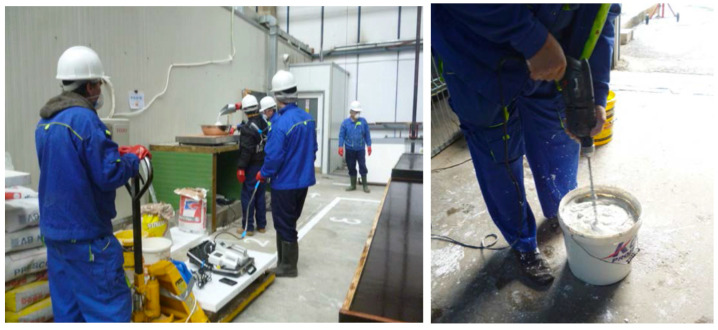
Raw material preparation.

**Figure 6:**
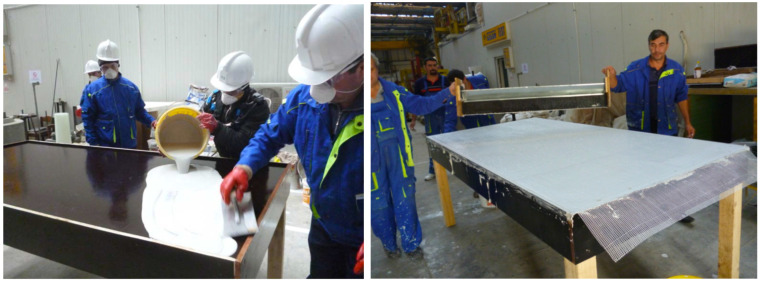
Manufacture of fire-resistant panel.

**Figure 7:**
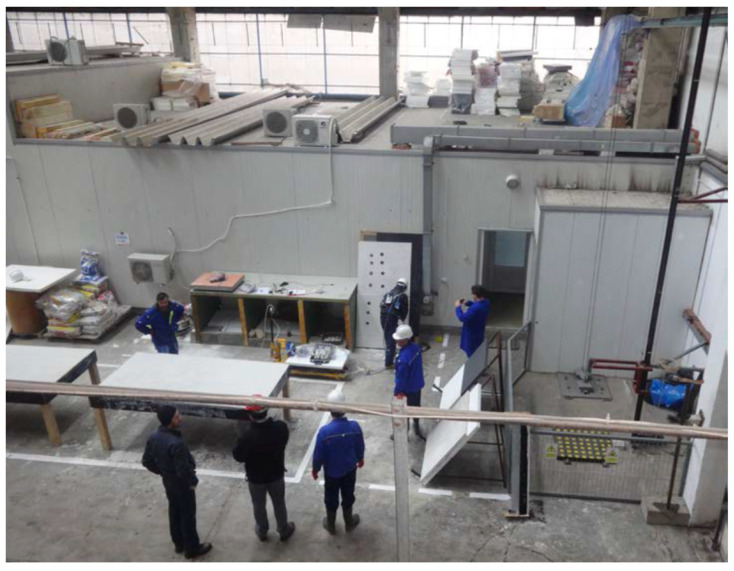
Finishing of panel and storage.

The manufacture of fire-resistant panels containing bentonite nanoclay, activated with sodium carbonate, is according to the following technological stages:

- Storage and mass dimensioning of raw materials: gypsum (IM 99, Adeplast SRL, Corlatesti, Romania), activated bentonite nanoclay (Bentoflux SA, Satu Mare, Romania), water, fiberglass mesh (Etex Building Performance SA, Bucharest, Romania); Preparation of compositions (gypsum + activated bentonite nanoclay + water). The composition preparation requires dosage of raw materials and mixing/homogenization of raw materials, Figure 5;
- Manufacture of fire-resistant panels (real dimension: 2300 × 1000 × 15 mm). Manufacture of panels requires lubrication of framework, casting of panels (applying the 1st layer of composition and leveling, applying the reinforcement layer of fiberglass mesh, applying the 2nd layer of composition and finishing), drying/hardening of panels, and de-molding of panels, as in Figure 6.
- Casting and finishing of panel; Storage of panels and aging—Racks/storage room, Figure 7.

**Figure 8:**
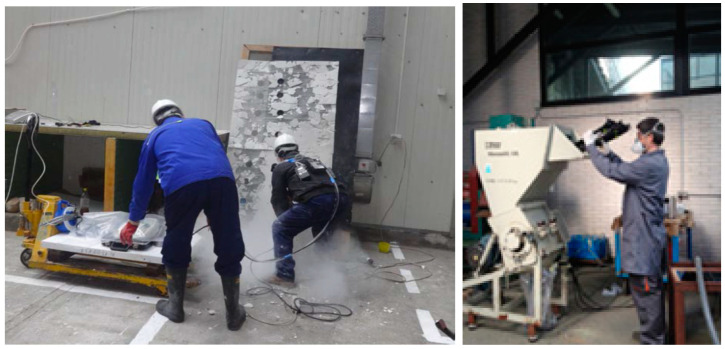
Panel post-processing by drilling and cutting; Recycling.

**Figure 9:**
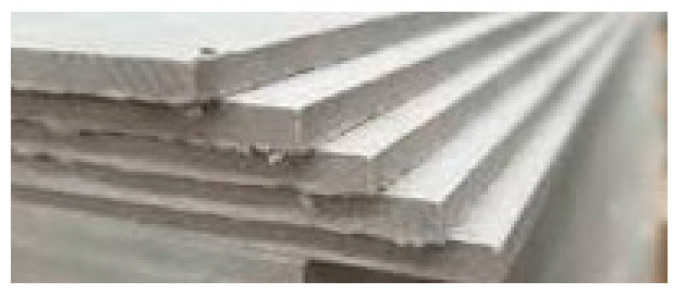
Example of commercial fire-resistant insulation panels containing nanoclay.

A demonstration of fire-resistant panels post-processing by drilling and cutting was also performed, Figure 8, followed by a recycling process by grinding. In Figure 9, an example of such a commercial product of a fire-resistant insulation panel containing nanoclay is presented.

The life cycle of a product has been separated into five key stages: (1) Design; (2) Technological operations for manufacture; (3) Embedding within construction with post-processing; (4) Maintenance and use; (5) Demolition and recycling. The critical steps for the nano exposure evaluation are 2, 3, and eventually 5 (similar in fact to exposure with 3), where measurements related to inhalation and dermal exposure were performed.

### 2.4. Experimental Measurements for Assessment of Inhalation Exposure to Nanopowders

According to ISO/TR 27628:2007 [40] and with the Manual of Analytical Methods [41], the recommended measurements are particle mass and number and surface area in a controlled environment. The measuring devices used for each measurement are described in Table 2, with their relevant features.

The performed measurements included the following: 1. Dusting test: Characterization of material release when handling powders of raw materials for the development of recipes containing nanoclays; 2. Mixing test: Characterization of the release of the material in the process of mixing the materials for the realization of recipes; 3. Panel setting: Characterization of material release during setting/hardening processes; 4. Accidental fire: Characterization of material release in accidental fire (along with smoke toxicity and opacity); 5. Panel post-processing: Characterization of material release by drilling and cutting (usual commercial electric tools were used, e.g., high strength steel drill HSS 8 mm diameter, speed of 1000 rpm); 6. Recycling: Characterization of material release by grinding/shredding (MATEU & SOLÉ 25/40 10M shredding equipment, Mateu y Solé SA, Barcelona, Spain). The experimental settings were established to simulate the different processes mentioned above. Specifically, the handing loads of a powder were simulated based on the dust tests recommended in ISO/TC 229 [42]. The measurement strategy followed a sequential orientation approach based on the NanoGEM project [43] (screening and advanced measurements). Accidental fire was evaluated in accordance with ISO 5660-1:2015 [44], along with nanoparticle measurement. The on-site measurements provided data regarding the release of particles from the processes. Nevertheless, customization of extrapolation is possible by considering workers’ activity patterns to offer guidance on best practices in the construction industry. The measurements were conducted close to where the worker was carrying out the particular task. There was no ventilation provided in the working space. The data are analyzed for a total of 3 consecutive readings, which are recorded 30 s after initiating the task once the emission levels are deemed stable (following the same assumption as in the theoretical model).

## 3. Results and Discussion

### 3.1. Inhalation Exposure Analysis

The findings regarding particle concentration are outlined in Table 3.

Compared to a state of ‘no activity’, each activity results in an exposure increased by a factor of min. 1000. Panel post-processing by drilling results in the highest exposure, followed by mixing and grinding tasks. The least amount of exposure occurred while workers were setting panels and working with the material in its fluid state. The unintended fire testing results were lower than anticipated, but this is offset by the fact that we tested fire-resistant panels with reduced smoke output. The setting process showed the most consistent data with minimal variability. Similarly, during grinding, emissions were steady and mainly originated from the material entering the grinder’s narrow space, directing particles toward the operator. Regarding the process of drilling or cutting, the standard deviation is elevated due to the tools used creating localized ventilation of released powders near the panel contact, resulting in emissions values that are more widely spread but consistently high.

Upon comparing the simulated data to the actual measured data, we observed that they specifically align within the range of 10^10^ particles/m^3^. Residual emissions in the working space during ‘no activity’ are minimal and do not significantly impact measured values during building operations, similar to simulation data without a residual initial value.

**Figure 10:**
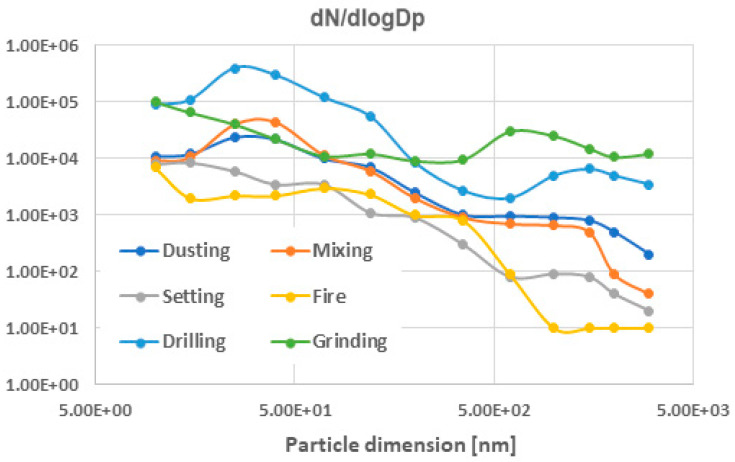
Size distribution of particles vs. test scenarios.

Regarding the distribution of particle sizes, we looked at the aerosol data, which followed a lognormal distribution, and computed the normalized concentrations dN/dlogDp, as referenced in [45]. Figure 10 displays the findings. It is evident that certain activities, such as drilling and grinding, release high amounts of emissions containing particles of various sizes ranging from 10 nm to over 2500 nm due to the mechanical abrasion of tools. The emissions from the fire test were primarily in the nanoscale range, reaching up to 500 nm. The manufacturing processes also resulted in emissions containing a wide range of particle sizes, but there was a significantly higher concentration of particles smaller than 1000 nm, particularly those smaller than 100 nm.

**Figure 11:**
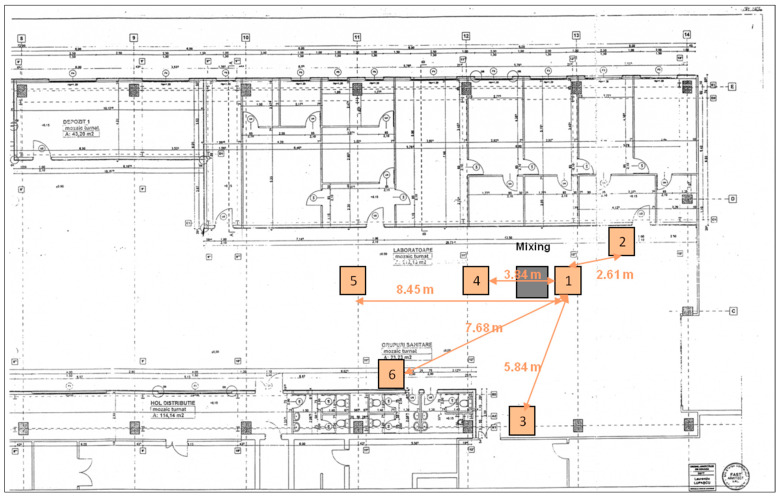
Placement of points for measuring particle parameters in the working area.

The second study involved mapping particle characteristics simultaneously at six points situated at varying distances from the nanoclay bentonite panel sample production site, as shown in Figure 11. Point 1 is the focus of the previous study, with the findings presented in Table 3. This time, only the ‘mixing’ step was selected, as it takes only 30 s and has the highest concentration of particles under 700 nm compared to other stages in manufacturing, as shown in Figure 10.

The data were gathered 4 min after the operation ceased to be compared with the outcomes from the software simulation. The mean concentration for the three tests conducted for every test is shown in Table 4. The size distribution analysis of particles was deemed unnecessary if assessed, as shown in Figure 10, with only the concentrations of particles expected to vary. The measurements were redone considering ventilation, with mechanical ventilation (exhaust system on) at point 4 and natural ventilation (open doors or windows) at points 2 and 3 having minimal impact. In all six points, the air velocity during the measurements varied in the range from 0.5 to 1.3 m/s, tests being made according to BS EN 12599:2012 [45]. At point 6, the air velocity exceeded 0.5 m/s. The highest air speeds were measured at point 1, up to 0.9 m/s, and at point 4, up to 1.3 m/s. At the beginning of the measurements, with ventilation off, the air humidity varied in six measurement points in the range of 36–41% and in the temperature in the range of 22.7–23.5 °C. When ventilation was turned on, air humidity decreased to 29–35%, and the temperature to 21.5–22.0 °C. The results are summarized in Table 4.

Overall, it is challenging to assess how the production process affects the changes in particle concentration in larger chambers’ air, especially when using ventilation, and to compare with simulations performed on a smaller scale. In the scenario ‘lacking ventilation’, the average results were approximately 5 times greater than the simulated values of 5.24 × 10^8^, which can be attributed to residual emissions in the work area that were not considered during the simulation. This discrepancy may also be influenced by the air speed of 0.1 m/s in the simulation when compared to the absence of air flow in actual conditions. When it comes to ventilation, the results were approximately 5 times lower than the simulation due to the larger size of the area and faster air flow. In any case, the ventilation in the actual scenario reduced the particle concentration by 20 times, which is still not considered satisfactory for effectively eliminating the nanoparticles.

There are two ways to measure the overall effectiveness of ventilation systems. One involves examining air velocities in the open state, which indicates how quickly air is removed in various locations depending on the activity and if it can eliminate different particles. The other method calculates exhaust air flow rates and air exchange rates in tested areas. The issue with these techniques arises when nanoparticles are present in the air flow and a filter is not available to effectively capture them, regardless of the air velocity. The actual dimensioning of ventilation systems is based on Equation (3), which evaluates the exhaust flow through the vents in m^3^/h:

(3) $$Q_{W}=3600\times F_{gross}\times v_{e,av}\times k$$

where $v_{e,av}$—average air velocity in the air outlet [m/s], $F_{gross}$—the gross surface area of the feed opening, *k*—coefficient that takes into account the air flow conditions at the outlet, with an average of 0.77 for the air flow between 0.5 and 1.3 m/s. If we apply the formula for the analyzed case, after measuring the air parameters (with Multifunctional air temperature/air humidity/air speed TESTO model 435-4, Testo AG, Titisee-Neustadt, Germany), we obtain the values from Table 5.

In theory, the ventilation method of ‘open door and turn on ventilation’ could meet the ventilation needs of the area by focusing solely on air circulation. However, as shown in Table 4, the concentration levels are still high. Moreover, even after the activity ceased and ventilation persisted for an additional 2 h, the concentration value only decreased by roughly 40%, highlighting significant concerns regarding the dangers of working with nanoparticles in open environments. During testing, nano-sized particles from all ventilation methods were released into the room air and detected in distant areas, reaching up to 17 m away from the source of exposure. Therefore, workers can only be safeguarded from nano-sized particles by using a proper ventilation system that includes air filters, which are highly effective. Our study tried to demonstrate the technical limitations of current ventilation systems, which are obvious when processing nanopowders, specifically nanoclays. On the other hand, it must be mentioned that on the use of actual filters and full-face masks [46,47,48] for protection from nano exposure, the results of the tests were inconclusive; the research in developing more efficient ventilation and protective systems must be intensified [49,50,51].

Ultimately, when evaluating the particle number concentration metric in all activities, it is necessary to compare those results with the nano-reference values established as safety limits. There is still debate over what the appropriate ‘safety’ level would be for each nanoparticle in any given situation. Many sources suggest that particles with a density less than 6 g/cm^3^, such as nanoclay, have a reference value of 40,000 particles/cm^3^. This value is specified as an 8-h average concentration, with the possibility of doubling for shorter time periods [52,53,54]. Based on the sources and the information in Table 3, in the scenario described earlier, the employees did not experience excessive exposure in the majority of the ‘manufacturing’ tasks over the specified brief timeframe. Analyzing the results, we note that it is possible to generally predict that adding nanoparticles to construction materials may not significantly raise workers’ exposure to nanopowders when considering particle concentration alone, compared to using traditional micro-scale materials. However, the issue still exists when it comes to blending powders or carrying out post-processing tasks for panels, where the concentration levels, such as during drilling, were ten times greater than for grinding and mixing powders, for example. 2.4 times greater than the reference, which is not tolerable, not even for very brief durations. Considering the findings from activities like drilling or disk cutting, nano exposure would be reduced by utilizing wet cutting or tools with powder collection systems. However, the most effective way to minimize nano exposure would be implementing an integrated automated processing line in closed areas with adequate ventilation systems, which is not always feasible in construction. In reference to the ventilation system cases in Table 4, all values seem to be below the accepted nano reference values. However, we do not consider working with nanopowders in this scenario safe. It is safe to enter the area where nanopowders were previously used.

### 3.2. Dermal Exposure Analysis

**Figure 12:**
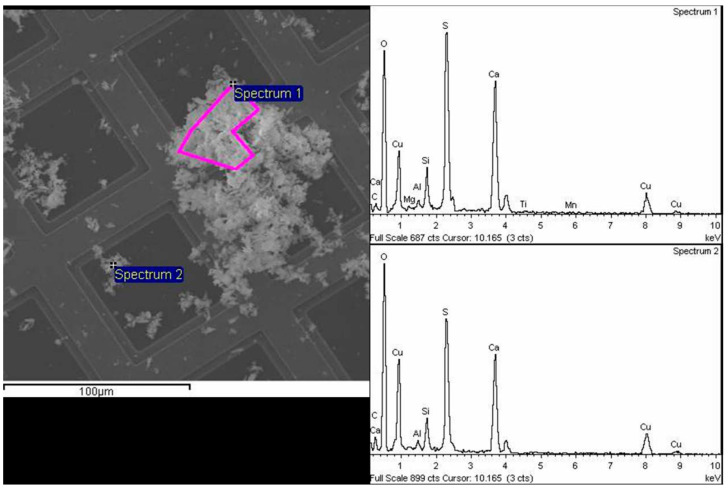
SEM analysis of powder samples.

Dermal exposure was also evaluated for the 6 test applications as presented above. The dermal exposure was evaluated using laboratory results (SEM/EDX) but was generally based on [55], in line with powder removal techniques [56]. The study included evaluating the gloves and masks worn by workers in various technical scenarios where skin and respiratory exposure measurements are required. The experiments conducted after 4 min of exposure in the specified activity areas indicated that there was no infiltration of nanoparticles through masks and gloves. However, there is still a widespread worry when taking into account longer durations of use, as well as the possible reutilization of costly masks [57]. At this time, the results obtained indicate that including nanoparticles in protective materials does not raise workers’ exposure to nanopowders compared to using traditional materials at a larger scale, considering particle concentration. In conclusion, it is important to mention that the measurements were conducted over brief durations and with small amounts of nanopartices, potentially leading to higher levels of uncertainty in the obtained outcomes. The measurements need to be repeated on an industrial scale to validate the alarming conclusions before moving on to larger-scale production. Samples from used gloves after drilling were analyzed at the laboratory level using SEM analysis, showing a high amount of nanoscale particles as in Figure 10, with chemical composition including gypsum (S and Ca) and clay (Si, Al, Mg), Figure 12. This indicates that gypsum, along with bentonite, contributes to nano exposure during certain technological processes. This aspect has not been considered for materials exposed at the micro-scale, which can be easily eliminated by ventilation or traditional protective measures.

In conclusion, a noteworthy point was made regarding the amount of powder that can accumulate on work gloves while engaging in various activities. By using an accurate analytical balance, it was demonstrated that the amount of nanopowder deposited decreased by approximately 38% with the ventilation system turned on. This raises additional doubts about the effectiveness and usefulness of ventilation systems when working with nanopowders in open areas. However, it is clear that the gathering of used equipment for destruction should take place in designated areas without ventilation.

### 3.3. Limitation of the Study and Perspectives

The research centered on nanoclays specifically, but its approach can be used for various other types of nanoparticles commonly found in the construction industry. The testing methods concentrated on examining inhalation and dermal exposure, yet there is potential to expand to consider ocular exposure or the lasting impact of nanoparticles on human health, as mentioned in [58,59]. The measurement techniques and tools were specially created to study the particle size, distribution, and surface structure of nanoclay particles, but they can be easily adapted for various experimental settings. The software simulation and industrial demonstration can be replicated for various industrial sites or materials technology with consistent databases, as mentioned in reference [60].

The study will continue to assess how well different ventilation choices and filters work, with regard to capturing nanoparticles, in order to offer new insights into improving worker safety in the construction industry. For this aim, in vitro evaluation of inflammation and toxicity can be established based on the frameworks outlined in references [61,62,63].

### 3.4. Safeguard Scenarios for Improving the Workers’ Safety

**Figure 13:**
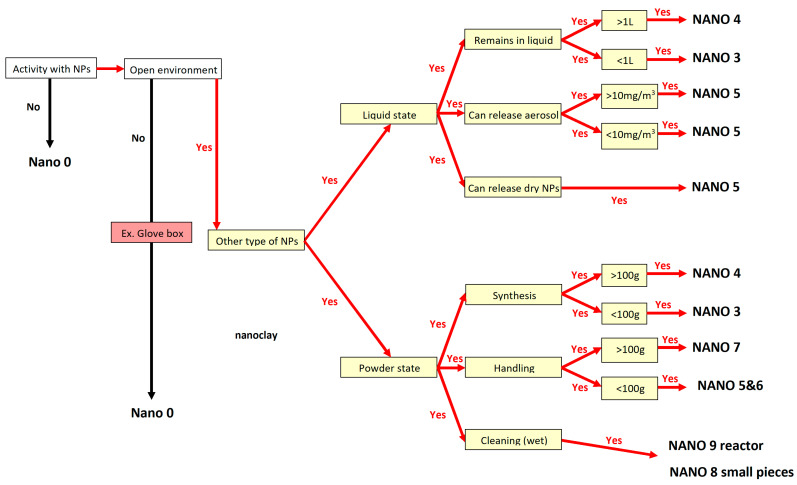
Decision tree for the nanoparticle contents that are less than 5%.

**Figure 14:**
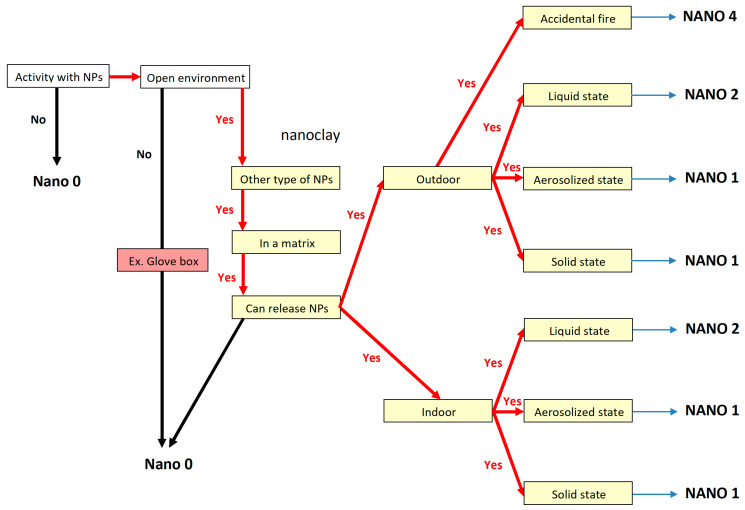
Decision tree for the nanoparticle contents that are more than 5%.

Given the high risk associated with nanoparticles, it is clear that new safety measures must be implemented to protect workers, especially in construction where open space exposure is greater [64,65]. A few situations were quickly examined, like in references [66,67], but lacked clear instructions for risk management and workplace safety, as current advice dictates [68,69,70,71,72]. The safeguard scenarios listed below align with safety suggestions and rely on specific decision trees, which should be matched with the potential nano exposure, necessitating an initial assessment of the active nanoparticles’ content (%) in workplace environments. Figure 13 and Figure 14 show two decision trees for nanoparticle content below or above 5%.

As a result, ten protection strategies have been created to guard against nanoparticle effects, presented as NANO 0–9, tailored to suit various technical situations for optimal protection:

- Nano 0: (1) No engineering control systems against nano hazards; (2) No respiratory equipment against nano hazards; (3) No dermal protection equipment against nano hazards.
- Nano 1: (1) General mechanical ventilation system with F7 + H14 air filters [47] OR natural ventilation by windows, doors, holes in walls; (2) FFP3 filtering half mask in all cases (indoor, outdoor) [48]; (3) Safety glasses, non-woven clothes like fleece jacket, one pair of nitrile gloves.
- Nano 2: (1) Natural ventilation by windows, doors, holes in walls, if indoor; (2) Full-face mask with a P3 filter in all cases (indoor, outdoor) [47,50]; (3) Safety glasses, chemical protective clothing cat. 3, one pair of nitrile gloves.
- Nano 3: (1) General mechanical ventilation system with F7 + H14 air filters OR natural ventilation by windows, doors, holes in walls; (2) FFP3 filtering half mask if powder state; (3) Safety glasses, lab. coat, one pair of nitrile gloves.
- Nano 4: (1) General mechanical ventilation system with F7 + H14 air filters OR natural ventilation by windows, doors, holes in walls; (2) No mask; if closed environment (reactor) -FFP3 filtering half mask, if open environment; Best fitted mask with powered filtering device incorporating a TH2 hood/full face mask with particle filter P3, if no collective protection, AND open environment; (3) Safety glasses, lab. coat, one pair of nitrile gloves.
- Safety glasses, chemical protecting clothing cat. 3, one pair of nitrile gloves, if accidental fire.
- Nano 5: (1) General mechanical ventilation system with F7 + H14 air filters AND fume cupboard; (2) FFP3 filtering half mask if no collective protection; (3) Safety glasses, lab coat, two pairs of nitrile gloves. Addition of safety sleeves if there is no collective protection.
- Nano 6: (1) General mechanical ventilation system with F7 + H14 air filters OR natural ventilation by windows, doors, holes in walls; (2) FFP3 filtering half mask in all cases; (3) Safety glasses, lab. coat, one pair of bi-colour rubber gloves.
- Nano 7: (1) General mechanical ventilation system with F7 + H14 air filters AND local exhaust system OR natural ventilation by windows, doors, holes in walls; (2) FFP3 filtering half mask if collective protection. Best fitted mask with powered filtering device incorporating a TH2 hood/full face mask with particle filter P3 (as in [48]) if no collective protection exists; (3) Safety glasses, lab. coat, two pairs of adapted nitrile gloves. Addition of a chemical protective clothing cat. 3, if no collective protection.
- Nano 8: (1) General mechanical ventilation system with F7 + H14 air filters AND fume cupboard; (2) FFP3 filtering half mask if collective protection. Best fitted mask with a powered filtering device incorporating a TH2 hood/full face mask with particle filter P3 if no collective protection; (3) Safety glasses, lab-coat, two pairs of adapted nitrile gloves.
- Nano 9: (1) General mechanical ventilation system with F7 + H14 air filters AND local exhaust ventilation; (2) FFP3 filtering half mask if collective protection. Best fitted mask with powered filtering device incorporating a TH2 hood/full face mask with particle filter P3 if no collective protection; (3) Safety glasses, two pairs of adapted nitrile gloves and chemical protective clothing cat. 3 in all cases.

The preventive purpose of the safeguard scenarios above is important to note and should be regularly updated for different types of nanoparticles or nanoclays, even if they appear similar or have similar health effects.

## 4. Conclusions

The article outlines the evaluation process of nano exposure in the construction industry, focusing on the commercial viability of producing fireproof insulating panels with nanoclay, which can be applied to other nanomaterials in the building industry.

An initial theoretical simulation of nanoparticle exposure in the workplace was conducted using the ANSYS CFX^®^ software. It was demonstrated that when there is no ventilation, diffusion becomes the primary factor for particle dispersion, and the decrease in concentration is gradual after the emissions stop. The manufacture stages for the fire-resistant panels containing bentonite nanoclay, which is activated with sodium carbonate, are described. The product life cycle has been divided into five main steps: (1) Design; (2) Technological operations for manufacture; (3) Embedding within construction with post-processing; (4) Maintenance and use; (5) Demolition and recycling. The critical steps for the nano exposure evaluation were 2, 3, and eventually 5 (similar in fact to exposure with 3), where measurements related to inhalation and dermal exposure were performed.

Panel post-processing determines the greatest inhalation exposure through drilling, mixing, and grinding actions. The workers experienced the least exposure when handling panels during the setting process as they primarily work with the material in its fluid form. The unintentional fire tests provided results below projections, but this can be offset by the fact that we tested fire-resistant panels with reduced smoke output. The setting process showed the most consistent data with minimal variation, while grinding results in all emissions coming from the material entering the narrow space of the grinder that directs particles toward the operator. In terms of the drilling/cutting procedure, the standard deviation is elevated due to the tools used causing localized dispersion of powders at the panel contact point, which results in consistently high but varied emission values.

Compared to a state of ‘no activity’, each activity resulted in an exposure increased by a factor of min. 1000. An overall assessment suggests that the use of nanoparticles in construction materials may not significantly heighten workers’ exposure to nanopowders when considering particle concentration alone, as opposed to using traditional micro-scale materials. However, the issue persists when blending powders or performing post-processing tasks for panels, with concentration levels being 10 times higher, for activities like drilling and grinding or mixing powders of min. 2.4 times more than the usual reference value, which is not permissible, even for brief durations.

In theory, with ventilation on and the door open, the space can be efficiently ventilated, but the concentration values still remain high. Furthermore, even after the activity ceased and the ventilation persisted for an additional 2 h, the measured concentration value only decreased by approximately 40%, thus raising significant concerns about the risks of working with nanoparticles in open environments. The research also aimed to highlight the lack of effectiveness of commercial ventilation systems when working with nanopowders, specifically with nanoclays. Many sources of literature emphasize the necessity of an update of ventilation standards when dealing with nanopowders, pointing out the shortcomings of current ventilation systems and, notably, the insufficiency of the associated filters.

The study of skin contact included the examination of gloves and masks worn by employees across various technical scenarios. Tests conducted after 4 min of exposure indicated that nanoparticles did not penetrate masks and gloves. Lab experiments demonstrate that both bentonite and gypsum contribute to nano exposure during specific technological processing—a factor not previously considered for materials known to have exposure at the micro level.

In conclusion, suggestions were made for enhancing worker safety, using decision trees as a basis. Therefore, ten protection strategies have been created to combat the impact of nanoparticles, formulated as NANO 0–9, which are tailored to provide optimal protection based on varying technical conditions. The safeguard scenarios were designed solely for preventive purposes and need to be revised for different nanoparticles, regardless of any potentially similar health effects they may cause. The specific decision trees may be further generalized for other specific technological processes in building areas by different end users.

## Figures and Tables

**Table 1:** Composition of bentonite nanoclay.

Formula	Z	Concentration	Stature	Line 1	Error.	LLD	Laser Analysis
SiO_2_	14	74.81%	XRF 1	Si KA1-HR-Tr	0.348%	222.3 PPM	13.8 μm
Al_2_O_3_	13	12.62%	XRF 1	AlKA1-HR-Tr	0.778%	224.1 PPM	11.1 μm
K_2_O	19	3.21%	XRF 1	K KA1-HR-Tr	1.06%	55.9 PPM	34 μm
Na_2_O	11	2.69%	XRF 1	Na KA1-HR-Tr	2.40%	444.5 PPM	4.7 μm
CaO	20	2.51%	XRF 1	Ca KA1-HR-Tr	1.25%	73.8 PPM	42 μm
MgO	12	2.29%	XRF 1	Mg KA1-HR-Tr	1.78%	365.0 PPM	7.4 μm
Fe_2_O_3_	26	1.58%	XRF 1	Fe KA1-HR-Tr	0.626%	31.0 PPM	181 μm
TiO_2_	22	0.25%	XRF 1	Ti KA1-HR-Tr	3.59%	53.6 PPM	68 μm
MnO	25	0.04%	XRF 1	Mn KA1-HR-Tr	5.25%	32.2 PPM	143 μm

**Table 2:** Measurement equipment.

Measured Parameter	Equipment	Domain
Number Surface area	AeroTrak-Plus Portable Particle Counter A100 (TSI Incorporated, Shoreview, MN, USA)	10–1000 nm
Mass Dimension	Dekati Low Pressure Impactor DLPI (Dekati Ltd., Kangasala, Finland)	30 nm–10 μm

**Table 3:** Particles number/m^3^ vs. 6 test scenarios.

Scenario	Mean Value	St. Dev.	Max Value	Min Value
No activity	5.96 × 10^6^	8.91 × 10^5^	6.95 × 10^6^	4.74 × 10^6^
Dusting	2.89 × 10^10^	1.56 × 10^10^	4.28 × 10^10^	1.16 × 10^10^
Mixing	9.89 × 10^10^	4.56 × 10^9^	1.04 × 10^11^	9.49 × 10^10^
Setting	4.58 × 10^8^	9.72 × 10^7^	5.02 × 10^8^	3.16 × 10^8^
Fire	7.66 × 10^9^	3.84 × 10^9^	1.24 × 10^10^	4.79 × 10^9^
Drilling	4.42 × 10^11^	1.54 × 10^11^	6.23 × 10^11^	3.15 × 10^11^
Grinding	9.62 × 10^10^	2.99 × 10^9^	1.01 × 10^11^	9.55 × 10^10^

**Table 4:** Particles number/m^3^ vs. 6 measuring points.

Point	Mean Value without Ventilation	Mean Value with Ventilation
1	2.24 × 10^9^	1.34 × 10^8^
2	2.11 × 10^9^	1.21 × 10^8^
3	2.02 × 10^9^	1.11 × 10^8^
4	2.08 × 10^9^	1.18 × 10^8^
5	1.77 × 10^9^	1.01 × 10^8^
6	1.92 × 10^9^	1.02 × 10^8^

**Table 5:** Ventilation efficiency in different conditions.

The Situation	Average Air Speed	Air Flow
	m/s	m^3^/h
Ventilation off and door open	0.05	68.75
Ventilation on and door open	1.30	1787.50
Ventilation on and door closed	1.20	1650

## Data Availability

The data are presented in this study.
